# Inflammation and vasopressin hypersecretion in aging

**DOI:** 10.3389/fendo.2025.1689787

**Published:** 2025-10-13

**Authors:** Kerim Mutig, Svetlana Lebedeva, Prim B. Singh

**Affiliations:** ^1^ Department of Pharmacology, Institute of Pharmacy, I.M. Sechenov First Moscow State Medical University, Moscow, Russia; ^2^ Scientific Center of Genetics and Life Sciences, Sirius University of Science and Technology, Sirius, Russia; ^3^ Department of Medical Elementology, Peoples’ Friendship University of Russia (RUDN University), Moscow, Russia; ^4^ Department of Biosciences, School of Medicine, Nazarbayev University, Astana, Kazakhstan

**Keywords:** vasopressin, antidiuretic hormone, cytokine, interleukin - 1 β, interleukin-6, inflammaging, microinflammation

## Abstract

Low-grade inflammation, both hypothalamic and systemic, sensitizes the neuroendocrine response to osmotic stimuli whose proximate cause is chronic underhydration common in older adults due to diminished thirst perception. These events drive persistent vasopressin (VP) release. VP exerts antidiuretic effects via renal V2 receptors and functions as a stress hormone through widely expressed V1a and V1b receptors. These latter actions are central to inappropriate activation of the hypothalamic-pituitary-adrenal axis observed in aging, as VP stimulates secretion of the adrenocorticotropic hormone. The resulting sustained elevations in circulating VP and cortisol contribute to metabolic, renal, and cardiovascular disorders that compromise health and lifespan in older individuals. This review reconciles the concept of microinflammation with recent molecular insights into hypothalamic osmosensitivity, proposing a model for the maladaptive hypersecretion of vasopressin in advanced age. This framework may inform the development of targeted interventions to normalize VP secretion, thereby mitigating the metabolic, cardiovascular, and renal diseases that disproportionately affect older adults.

## Impact of vasopressin signaling on aging

Aging is associated with neuroendocrine dysregulation manifesting by increased levels of circulating vasopressin (VP) in a significant proportion of older adults (1–8). Elevated plasma VP levels measured by its stable surrogate marker copeptin have been associated with enhanced risk of cardiovascular, metabolic, and renal diseases disproportionally affecting older people (9–12). Chronic underhydration of older adults due to blunted thirst perception or impaired renal water conservation constitutes an obvious trigger for sustained VP secretion since antidiuresis is the principal function of VP, also referred to as the antidiuretic hormone (8, 13). Exaggerated VP secretion in older adults may be also related to the low-grade systemic or hypothalamic inflammation typically developing during aging because the major pro-inflammatory cytokines including the interleukin 1β (IL-1β), IL-2, and IL-6 function as potent VP secretagogues (14–24). Apart from the antidiuresis, VP acts as a stress hormone contributing to activation of the hypothalamic-pituitary-adrenal (HPA) axis and exerting peripheral vascular and metabolic effects on the blood pressure and systemic glucose availability (25). While the antidiuretic action of VP is mainly mediated via the renal vasopressin V2 receptor (V2R), the stress-related hormone effects depend on the V1b (V1b) and to a lesser extent on the V1a receptor types (25, 26). Sustained HPA hyperactivity has been closely associated with aging pathophysiology in human and animals (27–31). VP-deficient Brattleboro rats and V1bR-knockout mice exhibit attenuated baseline HPA activity and blunted HPA response to various stressors suggesting a significant role of VP in the HPA activation with potential implications for aging (32–35). Peripheral effects of VP may provoke insulin resistance, hyperglycemia, vasoconstriction, hypertension, and renal damage at long term (9, 10, 25, 36, 37). Therefore, sustained VP hypersecretion may compromise health at multiple levels.

**Figure 1:**
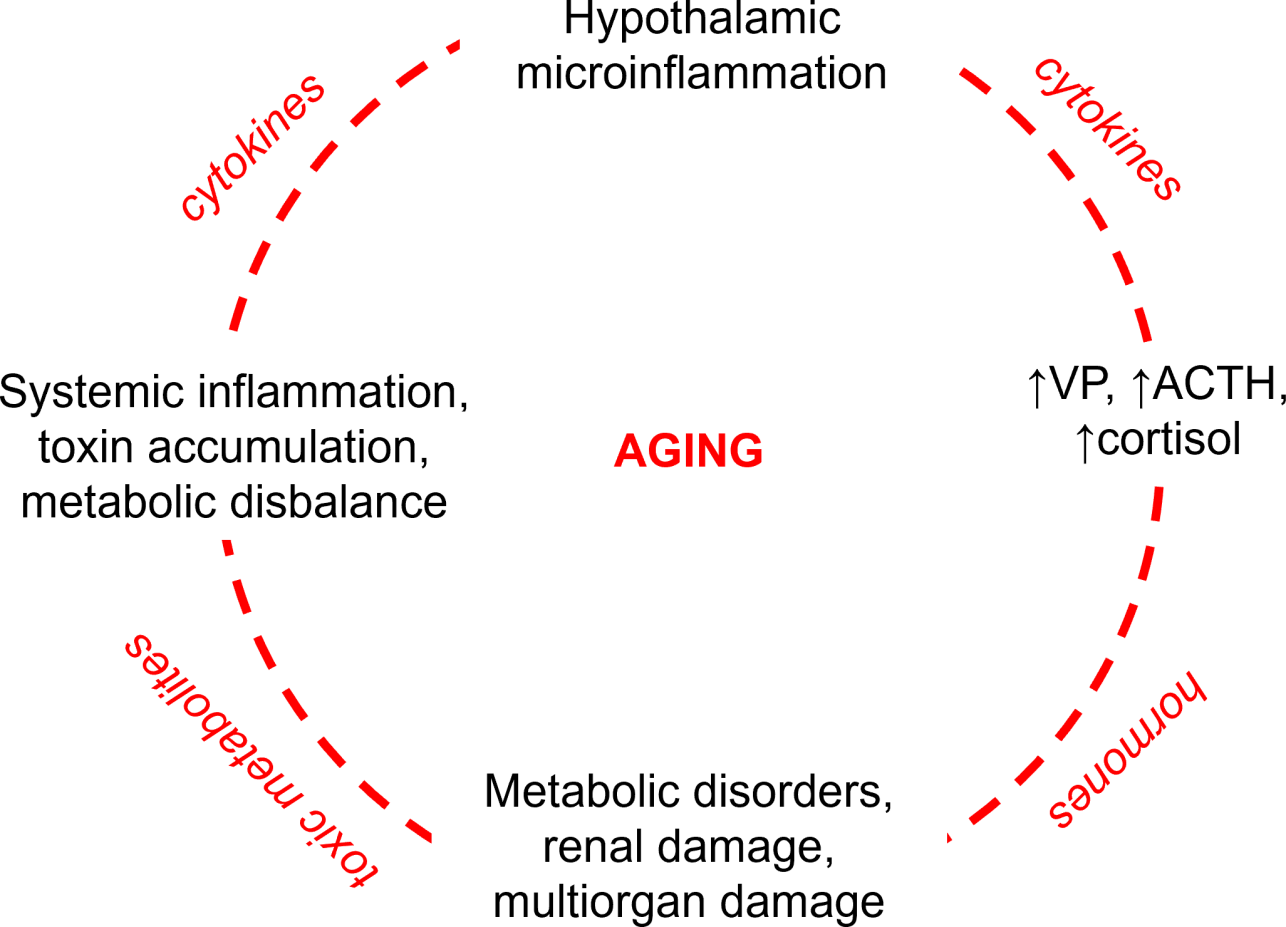
Vicious circle of pathophysiological events aggravating aging with focus on the role of vasopressin. Hypothalamic microinflammation induces sustained hyperactivity of the hypothalamic-pituitary-adrenal axis (HPA) enhancing central and peripheral secretion of vasopressin (VP) with ensuing sustained stimulation of the anterior pituitary producing the adrenocorticotropic hormone (ACTH) and adrenal glands producing cortisol. The resulting elevated levels of VP and cortisol provoke and aggravate systemic metabolic disorders such as diabetes mellitus, atherosclerosis, and hypertension with the ensuing renal and multiorgan damage. Impaired renal and cardiovascular performance lead to accumulation of toxic metabolites in the body and systemic inflammation. The latter aggravates the hypothalamic microinflammation by proinflammatory cytokines and toxic metabolites disrupting the brain-blood barrier.

In further sections we will outline potential triggers for exaggerated VP secretion with particular focus on the hypothalamic microinflammation and the low-grade systemic inflammation accompanying aging (14, 15) (**Figure 1**). We will consider a crosstalk between the osmotic and pro-inflammatory stimuli for VP release and integrate the recently identified molecular players in hypothalamic osmosensitivity to offer an updated model for maladaptive VP hypersecretion in advanced aging.

## Vasopressin biosynthesis and release in aging

VP is a hypothalamic-neurohypophyseal peptide hormone consisting of nine amino acids. Biosynthesis of the VP precursor takes place in magnocellular neurosecretory cells (MNCs) located within the supraoptic (SON), paraventricular (PVN), and accessory nuclei (AC) of hypothalamus and projecting to the posterior pituitary for hormone release into the peripheral blood (38). VP is also produced in a subset of hypothalamic parvocellular neurons which release the hormone into the hypophyseal portal circulation to potentiate the secretion of adrenocorticotropic hormone (ACTH) by the anterior pituitary (25, 39). The VP precursor (pre-provasopressin) contains VP at its N-terminus, the carrier protein neurophysin-2 in the middle, and a glycopeptide copeptin at the C-terminus (40). Proteolytic cleavage of the precursor results in secretion of VP and copeptin in equimolar amounts so that copeptin can serve as a surrogate marker of plasma VP levels (9). VP promotes antidiuresis thus preventing dehydration and playing the key role in water homeostasis (26). Accordingly, VP is secreted in response to increased plasma osmolality or reduced blood volume that occur during dehydration (26). Furthermore, secretion of VP increases in response to hyperthermia and certain pro-inflammatory cytokines. The thermal stimuli may enhance the osmosensitivity of MNCs thereby eliciting a greater VP release in response to smaller increments in plasma osmolality (41). Heat stress may further lead to activation of the hypothalamic-pituitary-adrenal (HPA) axis involving VP as a modulating hormone (25, 42, 43). Induction of VP secretion by thermal or inflammatory stress is at least in part mediated by pro-inflammatory cytokines such as IL-1β or IL-6 (24, 44). Inflammatory stress has been generally associated with stimulation of VP release (24). Thus, several non-osmotic triggers such as low blood pressure, hyperthermia, and inflammation contribute to the regulation of VP secretion in addition to its osmotic stimulation.

**Figure 2:**
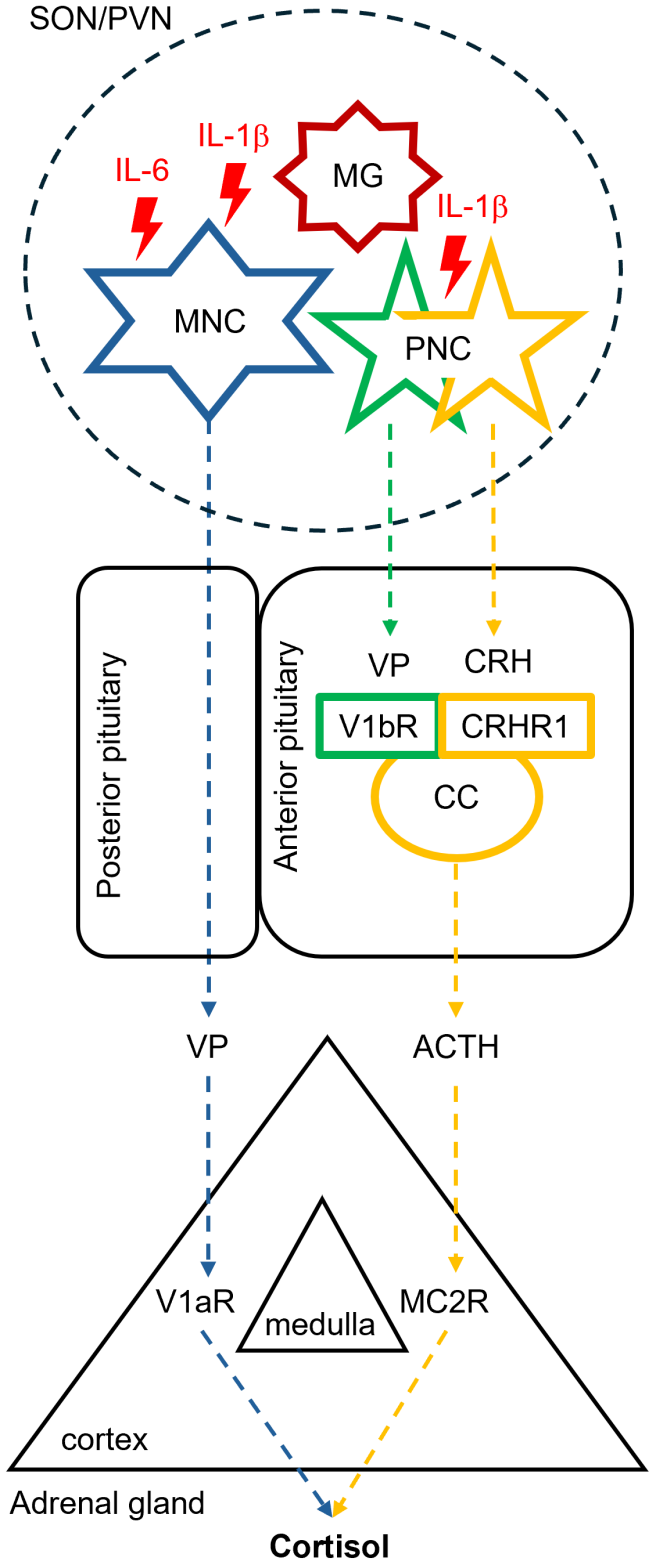
Putative effects of hypothalamic microinflammation on the neuroendocrine hypothalamic-pituitary-adrenal axis in advanced aging. Advanced aging is associated with enhanced hypothalamic release of pro-inflammatory cytokines including the interleukin 1β (IL-1β) and IL-6 by microglial cells. These interleukins enhance the excitability of neighboring vasopressin (VP)-producing magnocellular (MNC) and parvocellular neurosecretory cells (PNC) in the supraoptic (SON) and paraventricular hypothalamic nuclei (PVN). MNCs deliver an enhanced VP amount to the systemic circulation via the posterior pituitary, whereas PNCs release more VP along with the corticotropin releasing hormone (CRH) into the anterior pituitary. Activation of the corticotropin-producing cells (CC) residing in the anterior pituitary mediated by the vasopressin V1b receptor (V1bR) and CRH receptor type 1 (CRHR1) leads to enhanced secretion of the adrenocorticotropic hormone (ACTH). ACTH and VP synergistically stimulate cortisol secretion in the adrenal cortex via the melanocortin receptor type 2 (MC2R) and the vasopressin V1a receptor (V1aR), respectively.

The aging process has been associated with sustained increase of baseline plasma VP levels in a significant proportion of older adults (1–8). Hypothalamic nuclei retain largely intact morphology during aging but the neuroendocrine functionality of VP-producing MNCs is altered (45, 46). Post-mortem evaluation of hypothalamic regions in human brains from younger vs. older individuals revealed similar VP mRNA levels but increased VP-positive cell numbers and size in aged brains (47–49). Enlarged size of the Golgi apparatus in PVN and SON of older (over 70 years of age) compared to younger individuals suggested enhanced activity of MNCs in the aged human brain as well (50). Notably, nearly intact morphology along with structural correlates of high MNCs activity were observed even in brain samples derived from individuals with Alzheimer’s disease history (49, 50). Therefore, unlike the most other brain regions, hypothalamic nuclei are resistant to the aging-dependent neurodegenerative alterations but exhibit signs of increased activity instead. Chronic activation of MNCs in advanced aging may be triggered by the pro-inflammatory signaling arising from hypothalamic microinflammation (14). Experimental studies in transgenic animals link the hypothalamic microinflammation to neuroendocrine disbalance affecting health and life span (14, 51). Pro-inflammatory cytokines including IL-1β, IL-2, and IL-6 were established as potent HPA activators and triggers of the VP secretion (16–22, 52, 53). Stimulation of MNCs by these cytokines promotes sustained peripheral VP secretion via the posterior pituitary leading to elevated plasma VP levels. Parallel activation of vasopressinergic parvocellular neurons in PVN enhances VP delivery to the anterior pituitary, where it potentiates the effect of the corticotropin-releasing hormone (CRH) in V1bR-expressing corticotrophs thereby co-stimulating the adrenocorticotropic hormone (ACTH) secretion (35, 53, 54). Effects of ACTH are further supported by the V1aR-mediated co-stimulation of the adrenal cortex leading to enhanced cortisol secretion in response to ACTH, as reported by studies *ex vivo* and *in vivo* (42, 55–58). Thus, the exaggerated VP secretion driven by hypothalamic microinflammation appears to play a role in HPA hyperactivity during aging (**Figure 2**).

HPA activation alongside the VP hypersecretion may be also provoked by pro-inflammatory cytokines derived from the systemic circulation as reported for IL-1β or IL-6 in human and animals (21, 59). IL-6 plasma levels tend to increase during aging, and this cytokine plays the key role in development of the low-grade systemic inflammation in advanced aging also known as “inflammaging” (15, 60, 61). The reasons for elevated IL-6 plasma levels are likely multifactorial and may combine cellular senescence, dysregulation of immune system, and chronic diseases or metabolic conditions prevalent in older adults (15, 62). Although IL-6 or IL-1β cannot easily cross the intact blood-brain barrier (BBB) under normal conditions, the circulating cytokines may signal via circumventricular organs (CVO) placed outside the BBB thus affecting the neuroendocrine and behavioral functions such as thirst and VP secretion (63–67). In addition, gradual BBB disruption associated with aging may increase the exposure of VP-producing hypothalamic nuclei to the pro-inflammatory cytokines from systemic circulation (8, 15, 62, 68).

## Thirst vs. vasopressin secretion in aging

Adequate hydration of the body is critical to health. Physiological losses of water with urine, breath, sweat, and stool are balanced via behavioral adaptations driven by thirst sensation and ensuing water intake (69). The urine production by the kidneys constitutes the major route of daily water loss, disregarding atypical conditions such as fever or very high environmental temperatures resulting in extreme sweating (70). Intensive filtration of blood plasma in the kidneys is mandatory for rapid excretion of excessive or toxic water-soluble substances with the urine, whereas the ensuing tubular reabsorption of water prevents dehydration (71). Renal handling of water and electrolytes is under tight neuroendocrine control executed by VP (26). In advanced aging, water homeostasis is compromised both at the central and renal levels (72, 73). Blunted thirst sensation and impaired renal water conservation commonly occur in older adults leading to chronic underhydration and provoking sustained VP secretion (72).

Plasma hyperosmolality is the dominant stimulus for induction of both thirst and VP release, as the resultant intracellular dehydration is life-threatening and requires prompt normalization of the water homeostasis (26, 69). The osmosensory neurons triggering thirst reside in the forebrain regions known as the subfornical organ (SFO) and organum vasculosum of the lamina terminalis (OVLT), both regions belong to CVO placed outside of the blood-brain barrier and therefore directly exposed to changes in plasma osmolality (74). The osmotic stimulus for VP secretion originates from osmosensory SFO/OVLT neurons and converges with the intrinsic osmosensitivity of MNCs (75–77). The osmotic thresholds for thirst induction and VP release operate within a narrow range around 284–285 mOsmol/kg H_2_O in young healthy adults, as defined by several studies (8, 78, 79). Increments in plasma osmolality above that threshold range progressively trigger release of VP (~1 pg/ml per 1% osmolality) along with growing thirst perception (69, 80, 81). The close temporal association between VP release and thirst induction in young healthy adults promotes rapid rehydration with ensuing normalization of plasma osmolality and suppression of VP secretion.

Aging has been associated with blunted thirst perception but increased osmotic sensitivity towards VP release (8, 45, 82–84). The reasons for impaired thirst perception in older individuals are multifactorial including the low-grade systemic inflammation, since SFO and OVLT lack the blood-brain barrier and are directly exposed to the circulating pro-inflammatory cytokines and chemokines (8, 15, 62, 72, 85). In this context, administration of human IL-1β has been shown to suppress osmotic thirst in rats (66). Likewise, administration of the tumor necrosis factor (TNF) has been shown to reduce fluid intake in mice (86). However, these results were obtained with relatively high cytokine doses and thus model the acute inflammatory response rather than the low-grade inflammation in advanced aging. The available data on effects of pro-inflammatory cytokines on thirst perception in aging is still scarce. A recent study in aged individuals reported an inverse correlation between the skin hydration status and plasma levels of several pro-inflammatory cytokines including TNF, IL-1α, IL-1β, IL-6, and interferon γ (87). Although the topical water content in the skin partially reflects the global water homeostasis, serum osmolality is a more precise indicator of the body hydration status. Evaluation of serum cytokine profiles in healthy young vs. older adults showed no correlations between serum osmolality and circulating levels of IL-1β, IL-6, or TNF in one study (88). In view of the scarcity of data, further studies are awaited to clarify links between inflammaging and underhydration. Independently on the underlying mechanisms, impaired thirst perception and resultant chronic underhydration with moderately enhanced plasma osmolality persist in a significant proportion of older adults constituting an osmotic trigger for VP hypersecretion (2, 3, 5–8, 89–91).

## Pro-inflammatory cytokines and vasopressin secretion in aging

The physiological task of hypothalamic cytokine signaling is to prime the response of MNCs to stress during temporary perturbations of homeostasis (17, 92). In contrast, sustained exposure of hypothalamic tissue to proinflammatory cytokines such as IL-1β, IL-6, or TNF may provoke maladaptive morphological and functional synaptic remodeling of MNCs resulting in their enhanced sensitivity and exaggerated response to osmotic stress in advanced aging (14, 45, 93). Such synaptic reorganization may be aggravated by chronic underhydration frequently occurring in aged individuals (94). IL-1β is the key cytokine adjusting the MNCs excitability as its local release into SON in response to osmotic stimuli accompanies VP secretion (92, 95). Both hypothalamic neuronal and microglia cells serve as IL-1β sources, whereas the functional IL-1 receptor type 1 (IL-1R1) is present in neuronal cells such as MNCs or vasopressinergic parvocellular neurons but absent in microglia cells (52, 53, 92, 96). The major signaling pathways downstream of IL-1R1 include the Nuclear Factor kappa B transcription factor (NF-kB) and Mitogen Activated Protein Kinase (MAPK). Binding of IL-1β to IL-1R1 triggers the canonical NF-kB signaling via inactivating phosphorylation of the NF-kB inhibitor (IkB) provided by the IkB kinase (also known as IKK) (97). The ensuing nuclear translocation of NF-kB drives expression of target genes, including interleukins and enzymes involved in prostaglandin biosynthesis, which amplify the initial IL-1β effect on MNCs and parvocellular VP neurons via autocrine and paracrine mechanisms (52). Prostaglandins, in particular the prostaglandin E2 (PGE_2_), affect several ion channel types via modulation of cytosolic cAMP or Ca^2+^ levels with the net effect of increased MNCs excitability (98). IL-1β-induced activation of MAPK signaling may amplify intracellular Ca²^+^ signals critical for vesicular VP release (95, 99). Stimulation of the p38-MAPK kinases may also promote the VP mRNA expression via phosphorylation of the cAMP response elements (CREB) and activator protein 1 (AP-1) (100). Hypothalamic effects of IL-1β may be potentiated by induction of the IL-6 production and release (101). IL-6 expression in PVN and SON is induced by dehydration and the cytokine is secreted by the posterior pituitary parallel to VP to support metabolic adaptations to the dehydration stress (102). Administration of recombinant IL-6 to healthy volunteers or cancer patients has been shown to stimulate VP, ACTH, and cortisol secretion suggesting that peripheral IL-6 affects the HPA axis and VP secretion in human (21, 103). Stimulation of the IL-6 receptor (IL-6R) in MNCs promotes VP secretion via the Mitogen-Activated Protein Kinase/Extracellular Signal Regulated Kinase (MAPK/ERK) kinase cascade (104). Activation of the cyclooxygenase 2 (COX-2) and induction of PGE2 synthesis is integrated in both IL-1β and IL-6 signaling pathways and stimulates VP secretion via prostaglandin E2 receptors expressed in the hypothalamus (105, 106). TNF may modulate VP secretion directly or via complex interactions with the endocannabinoid system (ECS) (107–109).

**Figure 3:**
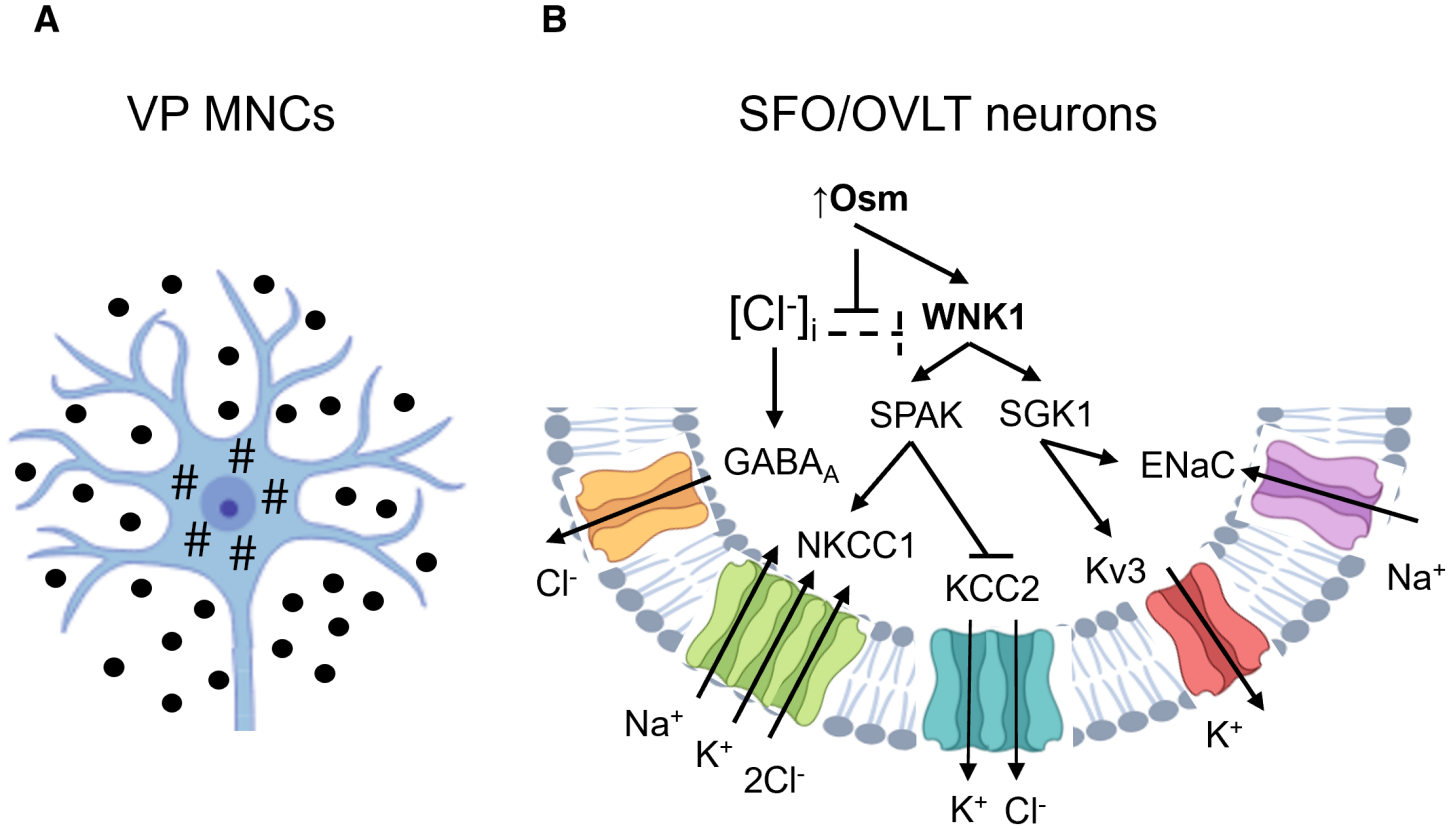
Putative molecular mechanisms mediating osmosensitivity in vasopressin-producing magnocellular neurosecretory cells **(A)** and osmosensory circumventricular neurons **(B)**. **(A)**: The response of vasopressin-producing magnocellular neurosecretory cells (MNCs) to hyperosmotic stress is mediated by the N-terminal transient receptor potential vanilloid 1 splice variant (ΔN-TRPV1), which activated by microtubular (#) condensation during cell shrinkage transmitting mechanical forces for the channel activation. **(B)**: In osmosensory neurons located in the subfornical organ (SFO) and organum vasculosum of the lamina terminalis (OVLT), hyperosmolality activates WNK1 in part by abrogating the inhibitory action of intracellular chloride ([Cl^-^]_i_). WNK1 signals via intermediate kinases, Ste20/SPS1-related proline/alanine-rich protein kinase (SPAK) and serum/glucocorticoid regulated kinase 1 (SGK1). The ensuing SPAK-mediated phosphorylation of the Na^+^-K^+^-2Cl^-^ cotransporter type 1 (NKCC1) and K^+^-Cl^-^ cotransporter type 2 (KCC2) produces reciprocal effects on their activity resulting in increased [Cl^-^]_i_ and neuronal excitability in response to GABA. SGK1-mediated activation of the epithelial sodium channel (ENaC) promotes a depolarizing passive Na^+^ leak, whereas stimulation of voltage-gated potassium channels (Kv3) enables fast and precise neuron firing. Arrows point to activation, whereas T-shaped bars indicate inhibition. Created with BioRender.com.

Chronic overexposure of MNCs to the aforementioned pro-inflammatory cytokines likely occurs in advanced aging due to hypothalamic microinflammation and systemic inflammaging (14, 15). These cytokines have been reported to reduce the threshold for MNCs depolarization via effects on several ion channel types. IL-1β has been shown to stimulate osmosensitive non-selective cation channels thus leading to influx of Ca^2+^, Na^+^, and K^+^ and increased excitability of MNCs (52, 92, 110). Although the identity of these channels remains to be clarified, members of the transient receptor potential vanilloid family (TRPV) emerge as appropriate candidates (77, 111–113). A functional N-terminal TRPV1 splice variant (ΔN-TRPV1) expressed in MNCs has been identified as a stretch-inactivated channel relevant for the intrinsic osmosensitivity of MNCs (114). Mechanical forces occurring during hyperosmotic stress and cell shrinkage are likely transduced on ΔN-TRPV1 via its C-terminal interaction with tubulin (115) (**Figure 3A**). Activation of ΔN-TRPV1 and the resulting Ca^2+^ influx induce the membrane depolarization with ensuing exocytotic VP release (114). The microtubular remodeling upon hyperosmotic stress further promotes the exocytotic membrane insertion of ΔN-TRPV1-containing vesicles via activation of the phospholipase C delta 1 (PLCδ1), as shown in mouse and rat MNCs (116). Thus, the initial ΔN-TRPV1-mediated calcium influx appears to trigger a positive feedback loop via PLCδ1 to amplify the ΔN-TRPV1 activity and MNC excitability upon sustained osmotic stress (117). According to this mechanism, IL-1β may sensitize the VP-producing MNCs thus causing stronger VP secretion in response to smaller increments in plasma osmolality, as has been reported in older adults (6, 8). The excitability of MNCs may be further potentiated by IL-6 since its increased hypothalamic expression in aged rat brains was linked to exaggerated VP release in response to immune challenge (118). The underlying molecular pathways and ion channels remain to be specified but may involve TRPV members or paracrine modulation of gap junction proteins in the surrounding astroglia (118–120). IL-6 receptor (IL-6R) is expressed in astrocytes also expressing the connexin 43 (Cx43), a gap junctional protein affecting the VP release via astrocyte-dependent uptake of neurotransmitters and neuropeptides and release of gliotransmitters (120–122).

In addition to the effects on MNCs excitability, the pro-inflammatory cytokines may affect the upstream osmosensory SFO/OVLT neurons projecting to vasopressin-secreting MNCs, as excitatory effects of IL-1β and TNF were documented in rodent CVO neurons (65, 123). Like in MNCs, studies in rats showed that IL-1β promotes depolarization of SFO neurons by activation of non-selective cation channels (64). The sensitivity of SFO/OVLT neurons to hyperosmotic stress is at least partially mediated by the with-no-lysine kinase 1 (WNK1), which detects molecular crowding during cell shrinkage thus acting as an intracellular osmolality sensor (124). Recent studies in mice link WNK1 activation to the VP secretion in response to plasma hyperosmolality (76, 125). WNK1 may affect neuronal excitability by modulation of several ion channels and transporters via intermediate kinases such as the STE20/SPS1-related proline/alanine-rich kinase (SPAK), the oxidative stress responsive kinase 1 (OSR1), or the serum- and glucocorticoid-inducible kinase 1 (SGK1) (126, 127). SPAK and OSR1 are homologous kinases targeting members of the electroneutral cation-coupled chloride cotransporters (CCC) family (128). Electrophysiological studies in rodent neurons showed that the Na^+^-K^+^-2Cl^-^ cotransporter type 1 (NKCC1) acts as a chloride importer, whereas the K^+^-Cl^-^ cotransporter type 2 (KCC2) is the major chloride exporter in mature neurons (129). Their functional interplay determines the intracellular Cl^-^ concentration [Cl^-^]_I_, neuronal excitability, and the type of response to the γ-aminobutyric acid (GABA)-induced signaling, i.e. inhibitory or excitatory (130). Both NKCC1 and KCC2 are substrates for phosphorylation by SPAK/OSR1 with opposing functional effects: activating for NKCC1 but inhibitory for KCC2 (131, 132). Thus, the WNK1-SPAK/OSR1 signaling may lead to intracellular chloride accumulation (**Figure 3B**). Since GABA_A_ receptor is a ligand-gated chloride channel, high [Cl^-^]_I_ suppress an inhibitory while provoking an excitatory effect of the GABAergic signaling, depending on the neuron type. A tonic inhibitory effect of GABA-signaling on the VP secretion has been observed *in vivo* and in cell culture (133, 134). In contrast, other animal studies report an excitatory effect of GABA on the VP release especially under certain pathophysiological conditions such as sustained hyperosmotic stress, diabetes, or hypertension (135–140). This switch may be related to enhanced NKCC1 activity and suppressed KCC2 function in MNCs and/or osmosensory SFO/OVLT neurons (137–140). Since diabetes, hypertension, and enhanced plasma osmolality due to underhydration are prevalent in older adults, these factors are likely to contribute to VP hypersecretion in advanced aging. Notably, these pathophysiological conditions are generally accompanied by sustained increases in circulating IL-1β, IL-6, TNF levels (141–144).

Apart from SPAK/OSR1, WNK1 may affect VP secretion via SGK1 (126). WNK1 activates SGK1 thus preventing ubiquitination and degradation of the epithelial sodium channel (ENaC), whereas ENaC activity has been shown to reduce the threshold for MCNs depolarization in rodents (115, 126, 145). ENaC is further expressed in the osmosensitive SFO/OVLT neurons and may be involved in their activation upon hypernatremia (146). Studies in rodents and observations in human suggest that ENaC activity may contribute to enhanced VP secretion in response to high dietary salt intake (115, 147–151). High-threshold voltage-gated potassium channels (Kv3) belong to the downstream targets of the WNK1-SGK1 signaling as well (76, 125, 152, 153). Their WNK1-induced activation has been shown to support the repetitive firing in mouse osmosensory circumventricular neurons thus stimulating VP release via respective projections to MNCs (**Figure 3B**). IL-1β and IL-6 have been reported to stimulate ENaC in epithelial cells but the respective effects in neurons have received only minor attention so far (154, 155). Likewise, effects on these cytokines on Kv3 channels have not been extensively studied in the neuronal context.

## Circadian VP secretion in aging

Healthy young adults exhibit a diurnal VP secretion pattern resulting in higher circulating VP levels at night and lower hormone levels during the day (156). The normal circadian rhythm of VP secretion is blunted in older people (157). Thus, insufficient rise of plasma VP levels at night may be involved in nocturia frequently reported by older people (157). Impaired renal response to the hormone may contribute to nocturia as well (73). Furthermore, VP is an established inducer of the ACTH secretion, which in turn stimulates production and secretion of the cortisol and aldosterone (158). The blunted circadian pattern of VP secretion may secondarily affect the diurnal rhythm of aldosterone or cortisol secretion in older individuals (159). Aldosterone secretion is also controlled by the renin-angiotensin system (RAS) activity but this regulatory pathway exhibits a gradual dissociation in advanced aging (160). Intact circadian pattern of the HPA neuroendocrine axis is crucial to global body metabolism, performance, and ability to elicit adequate stress responses (29, 161). VP is critically involved in maintaining the circadian rhythmic via its central and peripheral effects (162). The age-related circadian rhythm flattening results in decreased diurnal peaks but enhanced basal secretion of VP and cortisol which have been increasingly recognized as pathophysiological factors underlying diverse metabolic, renal, and cardiovascular disorders such as diabetes mellitus, atherosclerosis, or chronic kidney disease (163, 164). Notably, IL-1β has been shown to disrupt the pancreatic circadian rhythm with implications in the pathophysiology of diabetes mellitus (165). IL-1β-induced deterioration of the circadian rhythm has been also reported in articular cartilage during osteoarthritis (166). Characterization of IL-6-deficient mice revealed a role of IL-6 in the regulation of clock genes and behavioral rhythms of rest and activity (167). IL-6 secretion follows a biphasic circadian pattern in healthy young adults and is involved in the sleep/awake rhythm in human (168). Chronically elevated plasma IL-6 levels in cancer patients were associated with blunted diurnal variations in HPA activity (169). Taken together, sustained overexposure of the vasopressinergic system to pro-inflammatory cytokines because of hypothalamic microinflammation of inflammaging may contribute to flattening of the circadian VP secretion pattern but this assumption needs further experimental verification.

## Therapeutic prospects for targeting VP signaling in aging

Elevated levels of circulating VP frequently occur in older adults because of chronic underhydration and maladaptive alterations in sensitivity and strength of the neuroendocrine response to hyperosmotic stress, the latter may be related to hypothalamic microinflammation and systemic inflammaging (8, 14, 15, 62). Excessive and prolonged VP signaling has been implicated in cardiovascular, metabolic, and renal diseases (9, 37, 170, 171). Lifestyle modifications such as regular physical activity, adequate hydration, and metabolic dietary therapies can prolong health and life span in older adults. Pharmacological approaches retarding aging processes have been actively investigated in the recent decades.

### Water supplementation

Water supplementation to provide an adequate water intake is the obvious step towards prevention of dehydration and its negative consequences such as chronic stimulation of the VP system in older people. Indeed, several studies documented reduction of circulating VP levels in adults of different ages and health status receiving daily water supplementation, as judged by evaluation of the surrogate VP marker copeptin (172–175). Moreover, an adequate hydration and reduction of plasma copeptin levels were associated with improved glucose metabolism (172, 174, 175). However, excessive water intake bears a risk of euvolemic hyponatremia in older individuals since the aged kidney is limited in its capacity to excrete water (176). Therefore, a balanced water and electrolyte intake and regular monitoring of blood electrolytes is recommended in older individuals.

### Physical exercise

Sedentary lifestyle during aging has been associated with increased incidence of cardiovascular and metabolic diseases such as hypertension, obesity, or diabetes, whereas regular physical activity mitigates these risks in part by suppressing the inflammaging (177, 178). Systematic aerobic training reduces the baseline levels of circulating IL-1β, IL-6 and TNF, i.e. the pro-inflammatory cytokines potentially contributing to sustained VP hypersecretion in advanced aging (178, 179). With respect to VP, physical exercise causes acute transient increases in plasma VP and copeptin levels both in younger and older individuals (180–182). These increases reflect the body response to acute physical stress and serve to maintain the water homeostasis. Although elevated VP/copeptin levels are typically accompanied by enhanced IL-1β and IL-6 levels during physical exercise, the exercise-induced VP release appears to be largely independent on these cytokines (181, 183). In contrast, resting VP levels remain unchanged during periods of systematic physical activity, as has been demonstrated in older men and women assigned to endurance exercise (184). Since regular physical activity exerts beneficial effects on the circadian rhythm in older individuals it is tempting to speculate that physical training may stabilize the circadian VP secretion pattern as well (185, 186). Taken together, regular, age-matched physical activity of moderate intensity represents a valuable non-pharmacological intervention promoting healthier aging (187). Habitual aerobic exercise has been shown to ameliorate the HPA hyperactivity in older individuals although the impact of VP herein remains to be clarified (188, 189).

### Targeting inflammation

Derangement of VP signaling in advanced age is closely related with microinflammation of the hypothalamic tissue and increased production of pro-inflammatory cytokines such as IL-1β and IL-6 (14). Both cytokines are non-osmotic VP secretagogues (17, 18, 21, 190). Peripheral IL-1β or IL-6 induction in response to inflammation affects VP release as well, since these cytokines are able to disrupt the blood-brain barrier permeability and penetrate into the hypothalamic region (17, 67). IL-6 plasma levels tend to increase with aging independent on confounding factors such as major inflammatory diseases, whereas the circadian pattern of IL-6 secretion is flattened in older adults (61, 168, 191). The low-grade systemic inflammation promotes cellular senescence and metabolic disorders in aging (61). A growing repertoire and increasing availability of clinically approved IL-6 signaling inhibitors hold promise for retardation of aging-associated systemic and hypothalamic inflammatory processes triggering the inappropriate VP secretion (192). The extending clinical data pool from older individuals receiving IL-6 inhibiting drugs for treatment of autoimmune and inflammatory disorders may shed light on the utility of this strategy to manage age-related dysregulation in the VP system. Apart from pharmacological interventions, regular physical activity, especially aerobic exercise, has been established as a potent anti-inflammatory strategy to retard inflammaging (187).

### Selective V1a or V1b receptor antagonists

While the urinary concentration depends on the renal V2 receptors, the unfavorable metabolic and vascular effects of VP in older adults are largely mediated by activation of the V1a and V1b receptors (25, 26, 36). Thus, antagonizing either V1a or V1b receptor could improve the body metabolism while preserving the antidiuretic VP action critical to adequate hydration. The therapeutic potential of V1a and V1b antagonists is burgeoning, although none of them has been tested in the clinical settings of aging and metabolic diseases (193). Conivaptan, a dual antagonist to the V1a and V2 receptor types, has been approved for correction of euvolemic and hypervolemic hyponatremia similar to the selective V2 receptor antagonist tolvaptan (194, 195). A selective V1a receptor antagonist balovaptan has been tested in patients with autism spectrum disorders (196). Selective suppression of the V1b receptor showed promising preclinical results in the field of neurologic stress-related disorders (197). Nevertheless, the available experimental data strongly suggests that targeting V1a or V1b signaling bears therapeutic potential for improved management of the aging-related pathophysiology (25, 36).

### WNK-SPAK inhibitors

In view of the newly established role of the WNK1-SPAK/OSR1 signaling in hypothalamic osmosensitivity, selective pharmacological interventions in this pathway bear potential to blunt the excessive VP secretion in advanced aging (125). Inhibitors of SPAK and WNK kinases have been developed and showed therapeutic potential in animal models of hypertension and cystic fibrosis, although their utility in human requires further validation (198, 199). WNK1 fulfils multiple functions in immune cell biology ranging from the cell volume and motility to the regulation of cytokine production and pyroptosis (200). WNK inhibitors have been shown to exert toxic effects on the natural killer (NK) cells which may limit their therapeutic potential due to increased risk of malignancy (201). The cytotoxic effects of WNK inhibitors on the NK cells are likely mediated by disruption of the WNK1-OSR1 signaling, whereas selective inhibitors of SPAK downstream of WNK1 may have milder toxicity (201). Interestingly, WNK-dependent immunologic effects involve the mechanistic target of rapamycin (mTOR), whereas mTOR inhibitors have been shown to promote health and longevity in various animal models of aging (202). The mTOR signaling has been further shown to mediate some cell biological effects of VP such as autophagy inhibition (203). Therefore, beneficial effects of mTOR inhibitors may be partially related to improved cell metabolism due to disruption of the VP signaling.

## Conclusions and perspectives

Increasing recognition of the hypothalamic microinflammation and systemic inflammaging as significant factors driving maladaptive neuroendocrine processes such as VP hypersecretion in aging opens new perspectives for targeted lifestyle and pharmacological interventions. Recent progress in identification of molecular networks governing the physiological VP secretion adds to the choice of potential candidates for pharmacological targeting of the VP system. The emerging solutions include anti-cytokine therapies, selective inhibitors of V1a or V1b receptors, and suppression of the WNK1-SPAK signaling.
