# Correction: Inflammation and vasopressin hypersecretion in aging

**DOI:** 10.3389/fendo.2025.1724342

**Published:** 2025-10-23

**Authors:** Kerim Mutig, Svetlana Lebedeva, Prim B. Singh

**Affiliations:** ^1^ Department of Pharmacology, Institute of Pharmacy, I.M. Sechenov First Moscow State Medical University, Moscow, Russia; ^2^ Scientific Center of Genetics and Life Sciences, Sirius University of Science and Technology, Sirius, Russia; ^3^ Department of Medical Elementology, Peoples’ Friendship University of Russia (RUDN University), Moscow, Russia; ^4^ Department of Biosciences, School of Medicine, Nazarbayev University, Astana, Kazakhstan

**Keywords:** vasopressin, antidiuretic hormone, cytokine, interleukin - 1 β, interleukin-6, inflammaging, microinflammation

Affiliation “Department of Pharmacology, Institute of Pharmacy, I.M. Sechenov First Moscow State Medical University, Moscow, Russia” was omitted for author “Svetlana Lebedeva”. This affiliation has now been added for author “Svetlana Lebedeva”.

Author “Svetlana Lebedeva” was erroneously assigned to affiliation “Scientific Center of Genetics and Life Sciences, Sirius University of Science and Technology, Sirius, Russia”. This affiliation has now been removed for author Svetlana Lebedeva.

The original version of this article has been updated.

